# Spontaneous Entrainment of Running Cadence to Music Tempo

**DOI:** 10.1186/s40798-015-0025-9

**Published:** 2015-07-14

**Authors:** Edith Van Dyck, Bart Moens, Jeska Buhmann, Michiel Demey, Esther Coorevits, Simone Dalla Bella, Marc Leman

**Affiliations:** 1 IPEM, Department of Arts, Music and Theatre Sciences, Ghent University, Technicum Blok 2, Sint-Pietersnieuwstraat 41, Ghent, Belgium; 2 EuroMov, Movement 2 Health Laboratory (M2H), University of Montpellier, 700 Avenue du Pic Saint Loup, Montpellier, France

## Abstract

**Background:**

Since accumulating evidence suggests that step rate is strongly associated with running-related injuries, it is important for runners to exercise at an appropriate running cadence. As music tempo has been shown to be capable of impacting exercise performance of repetitive endurance activities, it might also serve as a means to (re)shape running cadence. The aim of this study was to validate the impact of music tempo on running cadence.

**Methods:**

Sixteen recreational runners ran four laps of 200 m (i.e. 800 m in total); this task was repeated 11 times with a short break in between each four-lap sequence. During the first lap of a sequence, participants ran at a self-paced tempo without musical accompaniment. Running cadence of the first lap was registered, and during the second lap, music with a tempo matching the assessed cadence was played. In the final two laps, the music tempo was either increased/decreased by 3.00, 2.50, 2.00, 1.50, or 1.00 % or was kept stable. This range was chosen since the aim of this study was to test spontaneous entrainment (an average person can distinguish tempo variations of about 4 %). Each participant performed all conditions.

**Results:**

Imperceptible shifts in musical tempi in proportion to the runner’s self-paced running tempo significantly influenced running cadence (*p* < .001). Contrasts revealed a linear relation between the tempo conditions and adaptation in running cadence (*p* < .001). In addition, a significant effect of condition on the level of entrainment was revealed (*p* < .05), which suggests that maximal effects of music tempo on running cadence can only be obtained up to a certain level of tempo modification. Finally, significantly higher levels of tempo entrainment were found for female participants compared to their male counterparts (*p* < .05).

**Conclusions:**

The applicable contribution of these novel findings is that music tempo could serve as an unprompted means to impact running cadence. As increases in step rate may prove beneficial in the prevention and treatment of common running-related injuries, this finding could be especially relevant for treatment purposes, such as exercise prescription and gait retraining.

**Key Points:**

- Music tempo can spontaneously impact running cadence.
- A basin for unsolicited entrainment of running cadence to music tempo was discovered.
- The effect of music tempo on running cadence proves to be stronger for women than for men.

**Electronic supplementary material:**

The online version of this article (doi:10.1186/s40798-015-0025-9) contains supplementary material, which is available to authorized users.

## Background

Approximately 56 % of recreational runners sustain a running-related injury each year [1]. About 50 % of all running-related injuries occurs at the knee and is most often due to the inability of the lower extremity joints to adequately control the loads applied during initial stance [2–4]. A number of strategies designed to reduce loads to these joints have been suggested, with one of the most common ones applying an increased step rate. Subtle increases in step rate have for instance been shown to substantially reduce the loading to the hip and knee joints during running and may therefore prove beneficial in the prevention and treatment of common running-related injuries [5]. However, less is known about the specific strategies that can be employed to change step rate. In this study, a novel strategy using music as a tool to impact step rate is examined. The means by which music might serve as an adequate tool for manipulating running cadence is discussed below.

A great deal of runners exercise while listening to music. This should not come as a surprise, since music listening during sport activities is believed to capture attention [6], distract from fatigue and discomfort [7], prompt and alter mood states [8, 9], enhance work output [10, 11], increase arousal [12], relieve stress [13], stimulate rhythmic movement [14], and evoke a sense of power and produce power-related cognition and behaviour [15]. Simpson and Karageorghis [16], for instance, examined the effect of music on a 400-m sprint performance while controlling for pre-performance mood. It was shown that music resulted in better sprint performance compared to the no music control. In another study, Styns et al. [17] observed that participants walked faster with music than with metronome ticks, while Bood et al. [18] showed that time to exhaustion was significantly longer with acoustic stimuli than without when participants were asked to run to exhaustion on a treadmill. Results of studies such as these suggest that music could be applied to physical activities, such as walking or running, with a considerable positive effect.

The idea that music can serve as a strategy for coping with physical exertion has been linked to the *parallel processing model*, which focuses on the limited human attention capacity [19, 20]. This implies that the focus of an exerciser is shifted to external events in an effort to reduce the perception of neural exertion signals coming from the muscles, joints, and cardiopulmonary systems [21]. However, it appears that external musical cues can only be the focus of attention in the case of low-to-moderate physiological awareness and perceived exertion. When the workload becomes too high, the exerciser’s attention is typically shifted towards the painful or fatiguing effects of the exercise [19, 20, 22–24]. In general, music has shown to be most effective to exert ergogenic and distractive effects when it is used to accompany self-paced exercise [8, 25–27]. In addition, it is believed that particularly motivational music can successfully uplift mood state and increase work capacity [9, 28, 29].

Besides the motivational factor, exercise that is repetitive in nature is believed to benefit mostly from music that is synchronized with the tempo of the exerciser’s movements; endurance can be extended, and performers exercise at higher intensities when moving in synchrony with musical stimuli [29]. It has been suggested that this effect of synchronized music is due to its ability to reduce the metabolic cost of exercise by enhancing neuromuscular or metabolic efficiency [28, 30]. Regular corporeal patterns demand less energy to imitate, due to the lack of timely adjustments within the kinetic pattern but also because of an increased level of relaxation resulting from the precise expectancy of the forthcoming movement [31]. As such, a point of reference is created that is able to attract and swiftly entrain recurring motor pattern efficiency [30, 32]. Synchronization is typically understood as an intentional mechanism, which is highly task constrained [33]. Most previous research on the impact of synchronized music on exercise performance generally focused on instructed or imposed synchronization, e.g. [12, 16–18, 29]. However, it is also the case that synchronization can occur spontaneously [33]. Previous studies have highlighted the natural or spontaneous predisposition of humans to respond to rhythmical qualities of music [34, 35], but much less is known about the capabilities of exercisers, and especially runners, to spontaneously synchronize with the tempo of musical stimuli. Yet, spontaneous entrainment of one tempo with another is only believed to occur when the strength of the coupling is able to overcome possible contrasts in natural movement period or tempo [36]. For a given coupling strength, unintentional entrainment only occurs within a specific range of period differences, reflecting the system’s entrainment basin [33, 37–40].

The effect of music on repetitive endurance activities also depends on the specific tempo of the musical stimulus. Waterhouse et al. [41] revealed that cyclists’ covered distance, power, and pedal cadence increased when faster music was presented, while slowing down the music tempo resulted in decreases of these measures. Edworthy and Waring [8] explored the effect of music tempo (and loudness) on treadmill running and demonstrated that an increase in the tempo, and to a lesser extent the loudness of the stimulus, resulted in an increase in running speed. In the light of findings such as those described above, it is quite plausible that music tempo could also serve as a means to influence running cadence. And as a link between step rate and hip and knee joint loading has been established before [5], results of this study could be particularly relevant with regard to the prevention and treatment of running-related injuries.

The aim of this study was to validate the impact of music tempo on running cadence. We hypothesized that recreational runners would adapt their self-paced running cadence to imperceptible changes in musical tempi and, thus, entrain spontaneously with the music tempo. Furthermore, we believed that the degree of entrainment would decrease with increasing changes in music tempo and, thus, that a basin for unintentional entrainment of running cadence to music tempo exists. As it has been shown that unintentional coordination typically manifests as relative or intermittent coordination (i.e. movements are attracted to a 0 or 180° but are not phase locked) [36, 37, 42], rather than phase-locked steps, *entrainment* refers to the amount of steps taken in a tempo sufficiently close to the music tempo (max. 1 % difference between running cadence and music tempo). Besides, since previous research often reported better results for women compared to men regarding music-to-movement coordination [11, 43], we expected female participants to display larger levels of entrainment. Finally, as it has been demonstrated that only when physiological awareness and perceived exertion are relatively low that music can distract from fatigue and discomfort [19, 22], the relationship between the level of entrainment and the degree of perceived exertion was examined.

## Methods

### Ethics Statement

The study was approved by the Ethics Committee of the Faculty of Arts and Philosophy of Ghent University, and all procedures followed were in accordance with the statements of the Declaration of Helsinki. In addition, all participants signed a form to declare that they participated voluntarily; that they had received sufficient information concerning the tasks, the procedures, and the technologies used; that they had the opportunity to ask questions; and that they were aware of the fact that running movements were measured, for scientific and educational purposes only.

### Participants

To establish sample size, a power analysis for a repeated-measures design was conducted using G*Power 3.1.9.2 [44]. Based on the effect sizes reported in comparable studies [16, 18, 28], the analysis indicated that minimally 14 participants for an α of 0.05 and a power of 0.80 would be required. Sixteen healthy adult participants (nine females) took part in the study. The test group consisted of recreational runners with an average age of 22.25 years (SD = 2.14), a mean body mass of 66.56 kg (SD = 9.32), and an average height of 1.74 m (SD = 0.10), who reported to be fit to run about 10 km. The majority (62.50 %) had received musical training (Fisher’s exact test showed no significant association between gender and musical background, *χ*^2^(1) = 2.05, *p* = .30). All participants reported that running is an activity that forms a part of their lives, with varying degrees of frequency (12.50 % runs multiple times a week; 56.25 % runs about once a week; 31.25 % runs about once a month; 0 % runs about once a year or not at all). Of all participants, 50 % reported to typically train with music, 32.25 % generally runs without music, and 18.75 % runs both with and without musical accompaniment.

### Stimuli

Previous research indicated that the natural running cadence for recreational runners lies somewhere between 130 and 200 steps per minute (SPM) [45]. On that account, a music database consisting of songs in the tempo range of 130–200 beats per minute (BPM) was created. A group of 19 students from Ghent University, all recreational runners, were asked to provide a list of at least ten songs they believed to be motivational to run to. From that specific list of music, the database for the experiment was created. In total, 117 songs with a clear beat and correct tempo range were pre-selected (see Table 1). In the course of the selection process, it was verified that the tempo of each song remained stable throughout the entire track. Using Audacity software (http://audacity.sourceforge.net), intros without clear beats were cut from the stimuli. BeatRoot [46] was applied to track the beats of each song in order to ensure that only songs between 130 and 200 BPM were included, while ReplayGain was used to normalize perceived loudness and minimize possible imbalances in sound pressure level.

**Table 1:** List of musical stimuli

ID	Artist	Song	Label(s)	Year published	Tempo (BPM)
1	Epica	Illusive Consensus	Transmission	2003	132
2	Gregory Porter	On My Way to Harlem (Radio Edit)	Motema	2012	138
3	Interpol	Slow Hands	Matador	2004	139
4	The Supremes	I Hear a Symphony	Motown	1965	139
5	Van Halen	Ain’t Talkin’ ‘Bout Love	Warner Bros	1978	139
6	Combichrist	Electrohead	Out of Line/Metropolis	2007	140
7	dEUS	The Soft Fall	PIAS	2012	140
8	P!nk	Who Knew	LaFace	2006	140
9	Noisettes	Never Forget You	Mercury/Vertigo	2009	141
10	Rammstein	Benzin	Motor	2005	142
11	Royksopp	Tricky Tricky	Astralwerk/EMI	2009	142
12	Deftones	My Own Summer (Shove It)	Maverick/Warner Bros	1997	143
13	16 Horsepower	Outlaw Song	Jetset	2006	144
14	Coldplay	In My Place	Parlophone	2002	144
15	The Hickey Underworld	Future Words	PIAS	2009	145
16	ABBA	Waterloo (English Version)	Polar/Epic	1973	146
17	Steppenwolf	Born to Be Wild	Dunhill/RCA	1967	146
18	The Sisters of Mercy	Alice	Merciful Release	1982	146
19	School Is Cool	The World Is Gonna End Tonight	Not on label	2011	147
20	Tom Odell	I Know	Columbia/In the Name Of	2012	147
21	Trixie Whitley	Irene	Unday Records	2013	147
22	Aphex Twin	Flim	Warp/Sire/WEA	1997	148
23	Bruce Springsteen	Dancing In the Dark	Columbia	1984	148
24	Nneka	Heartbeat	Yo Mama’s Recording	2008	148
25	Alt-J	Breezeblocks	Infectious	2012	149
26	Marco Borsato	Ik leef niet meer voor jou	Polydor	1995	149
27	A Perfect Circle	Thinking of You	Virgin	2000	150
28	Editors	An End Has a Start	Kitchenware/FADER	2007	150
29	Florence and The Machine	Dog Days Are Over	Island	2009	150
30	Guns N’ Roses	It’s So Easy	Geffen Records/Interscope	1987	150
31	Katy Perry	E.T.	Capitol	2010	150
32	Pearl Jam	Lightning Bolt	Monkeywrench/Republic	2013	151
33	The Killers	Spaceman	Island/Vertigo	2008	151
34	Bloc Party	Flux	Wichita/Vice	2007	152
35	Elton John	Saturday Night’s Alright (For Fighting)	MCA/DJM	1973	152
36	P!nk	Are We All We Are	RCA	2012	152
37	De Staat	Sweatshop	Cool Green Recordings	2011	153
38	Ike & Tina Turner	Nutbush City Limits	United Artists	1973	153
39	Kings of Leon	Sex On Fire	RCA	2008	153
40	OutKast	B.O.B.	LaFace/Arista	2000	153
41	The Black Eyed Peas	Pump It	Interscope	2005	153
42	Massive Attack	Teardrop	Circa/Virgin	1998	154
43	Kaiser Chiefs	Never Miss a Beat	B-Unique/Universal	2008	155
44	Morphine	Honey White	Rykodisc	1995	155
45	The Pipettes	Your Kisses Are Wasted On Me	Memphis Industries/Cherrytree	2006	155
46	The Strokes	Juicebox	RCA	2006	155
47	Hooverphonic	Mad About You (Orchestra Version)	Columbia	2012	156
48	Nirvana	In Bloom	DGC	1991	156
49	The Van Jets	Ricochet	Belvédère	2005	156
50	Air	Surfing On a Rocket	Virgin	2004	157
51	Millencolin	No Cigar	Epitaph	2000	157
52	The Beach Boys	Surfin’ USA	Capitol	1963	157
53	Shaggy	Boombastic	Virgin	1995	158
54	Jones & Stephenson	The First Rebirth (Original Mix)	Prolekult	1994	159
55	Kings of Leon	California Waiting	RCA/HandMeDown	2003	159
56	Michael Sembello	Maniac	Warner Bros	1983	159
57	OutKast	Hey Ya! (Radio Mix Club Mix)	LaFace	2003	159
58	Beyonce	Halo	Columbia	2008	160
59	Birdman & Lil Wayne	Stuntin’ Like My Daddy (Street)	Cash Money/Universal	2006	160
60	Customs	Justine	Noisesome/EMI	2009	160
61	Mastodon	Spectrelight	Reprise/Roadrunner	2011	160
62	TNGHT	Higher Ground	Warp/LuckyMe	2012	160
63	P.O.D.	Alive	Atlantic	2001	161
64	Queens of the Stone Age	Little Sister	Interscope	2005	161
65	‘T Hof Van Commerce	Baes (Radio Edit)	Plasticine	2012	162
66	Black Sabbath	Paranoid	Vertigo	1970	162
67	Blondie	One Way or Another	Chrysalis	1978	162
68	Karate	Ice or Ground	Southern	2002	162
69	Moby	Feeling So Real	Mute/Elektra	1995	162
70	Orchestral Manoeuvres In the Dark	Electricity	Factory	1979	162
71	U96	Love Religion (Video Edit)	Guppy/Motor	1995	162
72	Wham!	Wake Me Up Before You GoGo	Columbia	1984	162
73	Bomfunk MC’s	Freestyler	Sony Music Finland/Epidrome	1999	163
74	Jamaica	Cross the Fader	Downtown	2011	164
75	Midlake	Antiphon	Bella Union	2013	164
76	Muse	Survival	Helium 3/Warner Music Group	2012	164
77	Sugababes	About You Now	Island	2007	164
78	Ella Fitzgerald	A-Tisket, A-Tasket	Golden Options	2008	165
79	Ike & Tina Turner	River Deep Mountain High	Philes	1966	165
80	Green Day	Boulevard of Broken Dreams	Reprise	2004	166
81	Pixies	Where Is My Mind	4 AD	1988	166
82	Rammstein	Mann gegen Mann	Universal	2005	166
83	Arctic Monkeys	Do I Wanna Know	Domino	2013	170
84	Chet Faker	I’m Into You	Opulent/Remote Control	2012	170
85	Joy Division	Disorder	Factory	1979	170
86	Panic! At the Disco	I Write Sins Not Tragedies	Fueled by Ramen/Decaydance	2005	170
87	Queens of the Stone Age	No One Knows	Interscope	2002	170
88	The All-American Rejects	My Paper Heart	Doghouse/DreamWorks	2002	170
89	Foo Fighters	The Pretender	Roswell/RCA	2007	172
90	Netsky	Love Has Gone	Hospital	2012	172
91	Paramore	Misery Business	Fueled by Ramen	2007	172
92	The Streets	Fit But You Know It	Locked On/679	2004	172
93	DJ Fresh	Hot Right Now (Radio Edit)	Ministry of Sound	2012	174
94	Interpol	A Time To Be So Small	Matador	2004	174
95	Kanye West	Homecoming (feat. Chris Martin)	Roc-A-Fella/Def Jam	2008	174
96	Rudimental	Waiting All Night (feat. Ella Eyre)	Asylum	2013	174
97	Kelis & Andre 3000	Millionaire	Virgin	2004	176
98	Technohead	I Wanna Be a Hippy	Mokum	1995	177
99	Komatsu	Comin’	Lighttown Fidelity	2011	178
100	Mo’ Horizons	Pe Na Estrada (Radio Edit)	Agogo	2008	178
101	Tony Bennett & Lady Gaga	The Lady Is a Tramp	Sony Music Entertainment	2011	179
102	One Direction	Kiss You	Syco/Columbia	2012	180
103	Red Hot Chili Peppers	Can’t Stop	Warner Music	2002	182
104	The Pointer Sisters	I’m So Excited	Planet	1982	184
105	Ok Go	Don’t Ask Me	Capitol	2002	186
106	Joan Jett & The Blackhearts	I Love Rock ‘N Roll	RAK	1975	188
107	Wheatus	Teenage Dirtbag	Columbia	2000	188
108	Absynthe Minded	Pretty Horny Flow	Abeille Musique	2008	190
109	Eminem	Berzerk	Aftermath Entertainment/Shady/Interscope	2013	190
110	Macklemore & Ryan Lewis	Thrift Shop (feat. Wanz)	Macklemore LLC/ADA	2012	190
111	Roxette	The Look	EMI	1988	190
112	Isbells	As Long As It Takes	Zeal	2009	197
113	Beyonce	Crazy In Love (feat. Jay-Z)	Columbia/Music World	2003	198
114	Rihanna	Pon de Replay	Def Jam	2005	198
115	Gorillaz	Stylo (Radio Edit) [feat. Mos Def & Bobby Womack]	Parlophone/Virgin	2010	200
116	Wallace Vanborn	Atom Juggler	PIAS	2010	200
117	Linkin Park	In the End	Warner Bros	2000	210

### Apparatus

Participants were equipped with two iPods (fourth generation), one attached at each ankle. Using the Sensor Monitor Pro application on the iPods, data from accelerometers and gyroscopes was streamed wirelessly at 100 Hz to the main processing computer. A Wi-Fi hotspot (TP-Link N750) with special 3-dB gain antennas for longer range was used for maintaining a stable connection between the computer and sensors. Some minimal jitter and lag in the data stream were neutralized using a 500-ms buffer before processing.

Incoming sensor data was processed by a customized version of D-Jogger [47], a music alignment framework that selects and tempo-adapts music to runners’ gait frequencies using kinematic sensor input (Additional file 1). Music tempi were manipulated using a phase vocoder, which time stretches music without pitch modification. D-Jogger was adapted to match the experimental protocol (detect running cadence, playback tempo-matched music to this reference, increase or decrease music tempo). The system logged all data and calculations in real time. Finally, the resulting auditory stimuli were sent back to the participant using a Sennheiser HDR130 audio transmitter (with a range of up to 100 m). The participant perceived the music through Sennheiser HD60 headphones connected to the transmitter (attached to the upper arm). The delay due to the wireless audio transmission was negligible.

### Experimental Procedure and Set-up

The experiment took place in the Flanders Sports Arena of Ghent, Belgium. In order to select motivational music adapted to each runner’s personal taste, participants performed the Brunel Music Rating Inventory 2 (BMRI-2) test [48] at the start of the experiment. In this test, they were asked to rate all items of the music database by answering six questions about the motivational aspects of each song. Each item referred to an action, a time, a context, and a target (e.g. “The rhythm of this song would motivate me during a running exercise”) [49]. Participants responded on a seven-point Likert scale anchored by 1 (“strongly disagree”) and 7 (“strongly agree”). Afterwards, participants filled out a questionnaire on personal background, music education, and sports training. At the same time, for each participant individually, the 20 songs that had obtained the highest scores during the BMRI-2 test were loaded into the D-Jogger system.

Subsequently, participants were equipped with the iPods, the wireless headphone, and the audio transmitter. Each participant was asked to run on a 200-m running track for four laps continuously, for 12 times. Participants were instructed to run at their own comfortable tempo. No information was distributed concerning the real purpose of the experiment, and all participants ran in solo conditions. After each set of four laps, a break of approximately 5 min was introduced to enable the participant to recover sufficiently. Meanwhile, they were asked to indicate how heavy the effort had been during the exercise. This was rated on a Rating of Perceived Exertion (RPE) Scale [50], ranging from 6 (“no exertion at all”) to 20 (“maximal exertion”).

To get acquainted with the experimental set-up, the first set of four laps consisted of a practice set during which no music was played. Each of the 11 following four-lap sequences consisted of (1) a lap without music, (2) a lap with tempo-matched music, and (3) two laps with tempo-changed music. In the first lap, the participant ran at his/her self-paced cadence without musical accompaniment. In the second lap, music with a tempo matching the cadence assessed during the final 20 s of the previous lap was played. The musical stimulus consisted of the song that obtained the highest score during the BMRI-2 test with a tempo that differed maximally 5 % from the running cadence of the participant. After the song was selected, its tempo was adjusted to exactly match the mean running cadence. Finally, during the third and fourth laps, the tempo of the music was adjusted according to one of the 11 tempo-changed conditions.

In each of the 11 four-lap sequences, a different condition was tested. During the two final laps with tempo-changed music, the music tempo was adjusted to either −3.00, −2.50, −2.00, −1.50, −1.00, 0.00, +1.00, +1.50, +2.00, +2.50, or +3.00 % of its original one, played during the second lap. This range was chosen since an average person can distinguish tempo variations from about 4 % [51] and since the aim of this study was to test spontaneous or unintentional entrainment. The different conditions were randomized over the experiment in such a way that each participant performed all conditions but no participants performed the conditions in the same order. To ensure that they were not aware of the actual objective, participants filled out a questionnaire regarding their perception of the purpose of the experiment at the end. Responses did not indicate that they were aware of the experiment’s real purpose.

### Data Analysis

#### Cadence Adaptation

Running cadence was calculated using the iPods’ acceleration data. In order to check the degree of cadence increase/decrease, running cadence (SPM) recorded during the laps with tempo-changed music (*tempo-changed laps* or TCL) was compared to the cadence captured during the lap with tempo-matched music (*tempo-matched lap* or TML) and will be further referred to as *cadence adaptation*. As the tempo was gradually shifting during that period, the first 5 s of the laps with tempo-changed music was discarded. The final 20 s of those laps was also ignored as participants possibly altered their running behaviour due to the anticipated ending of the final lap (e.g. slowing down or speeding up).

$$ \mathrm{Cadence}\ \mathrm{adaptation}\ \left(\%\right) = \frac{\mathrm{avg}\left(\mathrm{S}\mathrm{P}\mathrm{M}\_\mathrm{T}\mathrm{C}\mathrm{L}\right)}{\mathrm{avg}\left(\mathrm{S}\mathrm{P}\mathrm{M}\_\mathrm{T}\mathrm{M}\mathrm{L}\right)} $$

#### Entrainment

A second measure of interest concerned the percentage of tempo-entrained steps during the laps with tempo-changed music. A step taken in a tempo sufficiently close to the music tempo (max. 1 % difference between SPM and BPM) at that specific moment is regarded as a tempo-entrained step. The tempo entrainment score is the percentage of tempo-entrained steps of the total amount of steps.

## Results

### Running Cadence

**Fig. 1:**
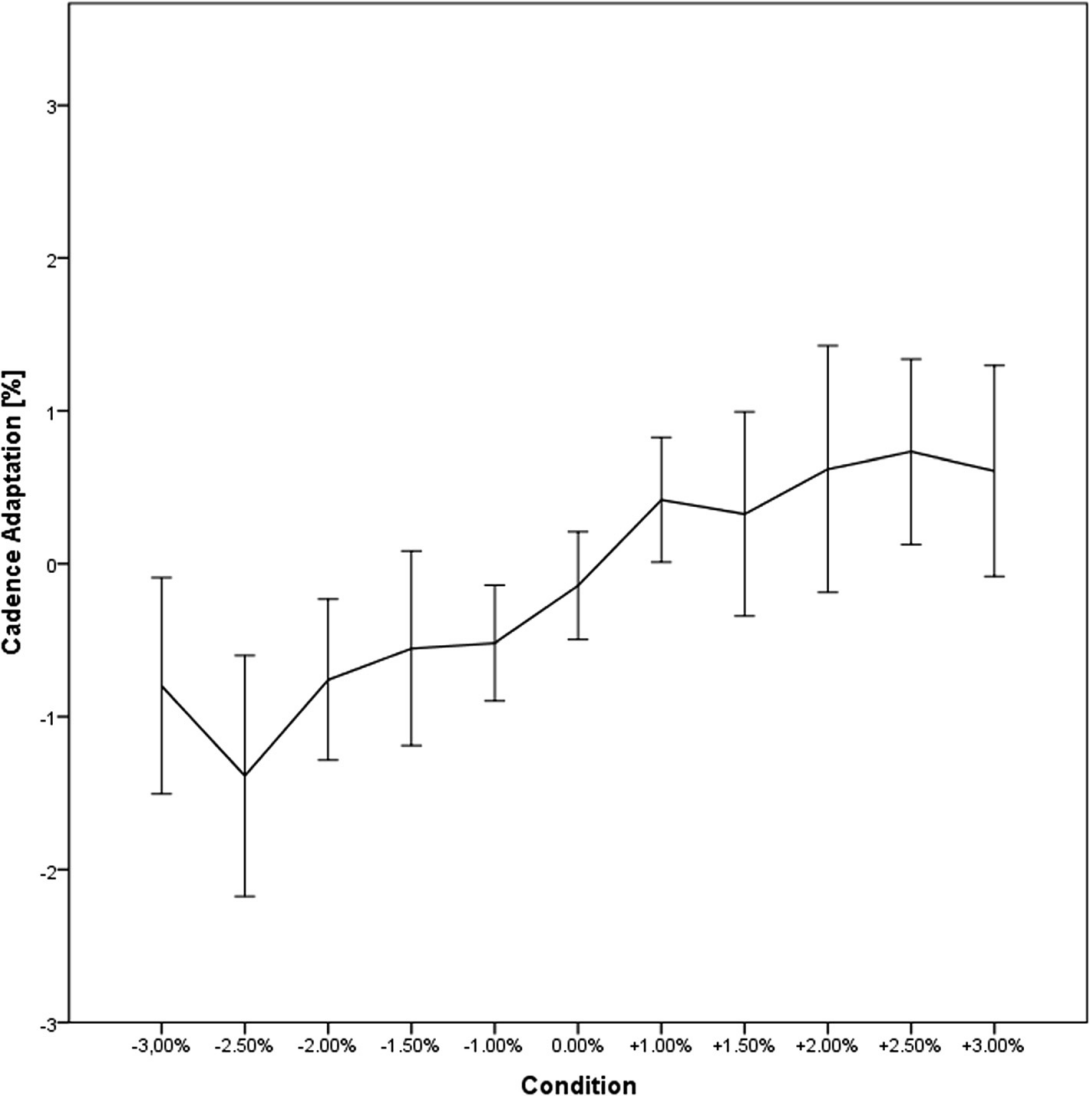
Mean tempo and cadence adaptation for the different conditions. Data presented is mean ± SE

This study tested whether the changes in music tempo would affect running cadence. A Kolmogorov-Smirnov test (KS test) showed that the assumption of normality was met, *D*(161) = 0.04, *p* > .05. A 11 × 2 × 2 repeated measures ANOVA with tempo condition as within-subject factor and gender and musical training as between-subject factors revealed a significant main effect of condition on cadence adaptation, *F*(10, 40) = 6.50, *p* < .001. Contrasts revealed a linear relation between condition and cadence adaptation, *F*(1, 4) = 94.56, *p* < .001, *r*^2^ = .96. The evolution of cadence adaptation over the different conditions is shown in Fig. 1.

**Fig. 2:**
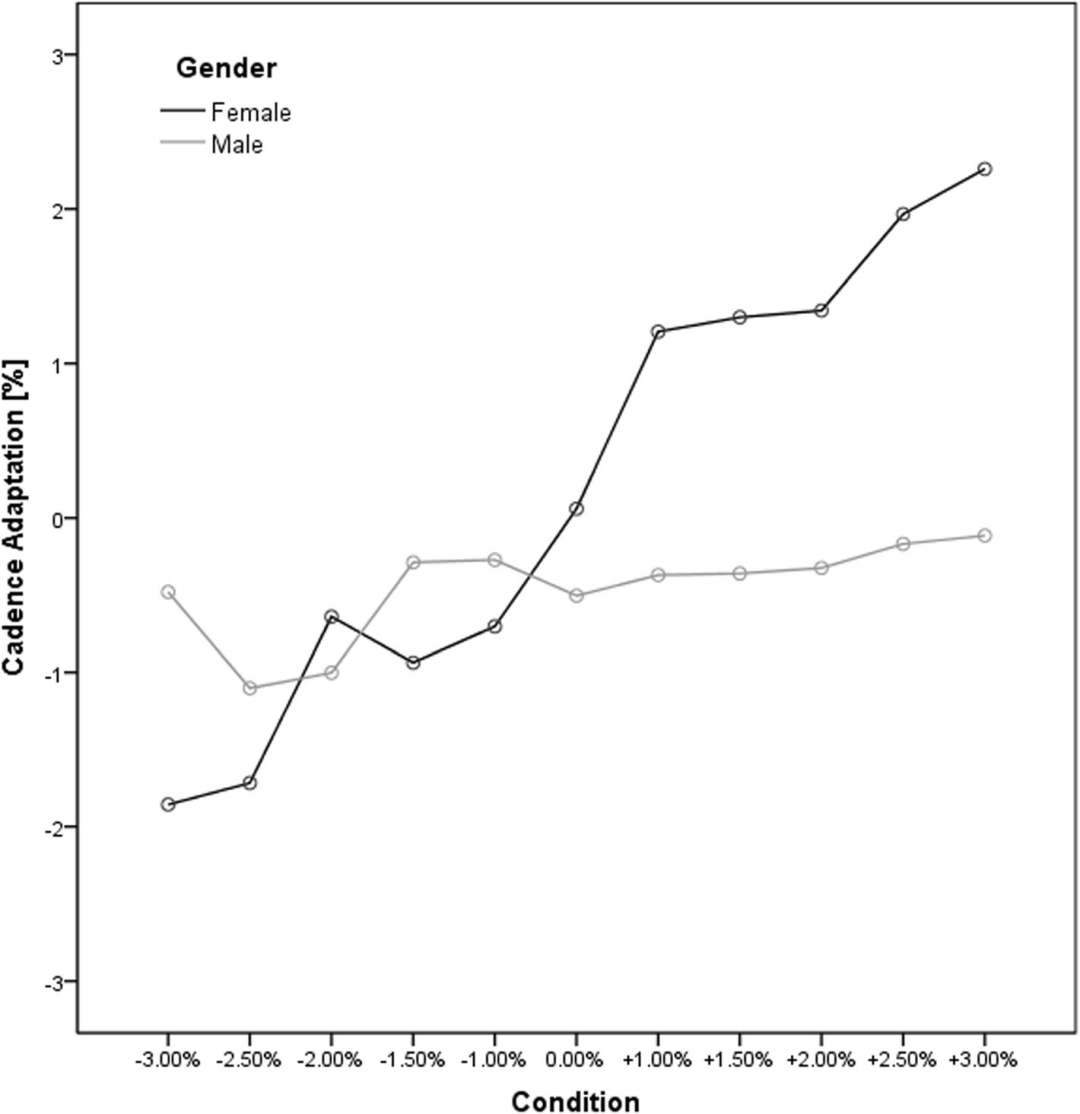
Interaction plot of estimated marginal means calculated for cadence adaptation at both gender levels

**Fig. 3:**
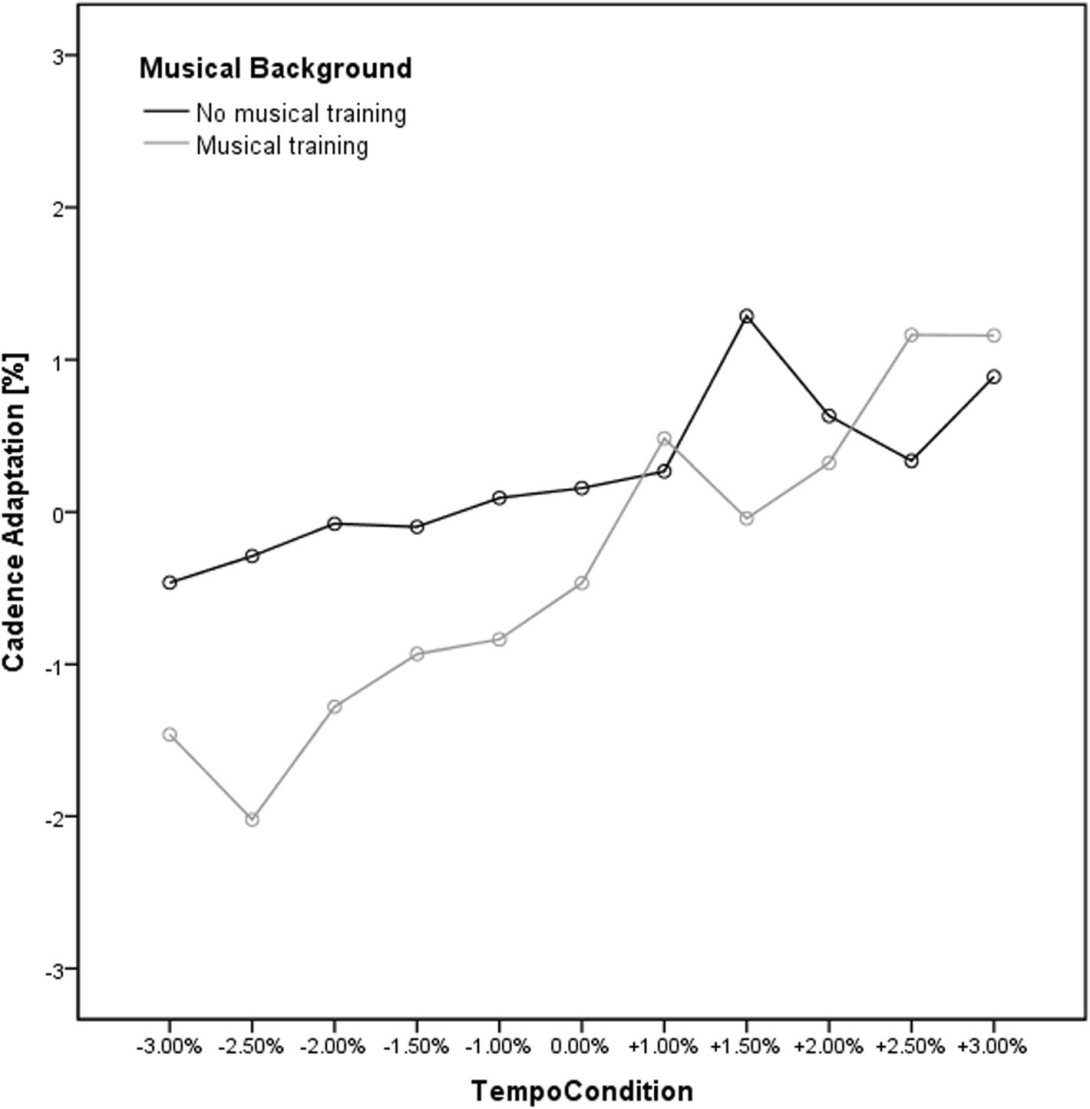
Interaction plot of estimated marginal means calculated for cadence adaptation at both musical background levels

There was no significant effect of gender, indicating rather similar levels of cadence adaptation for males and females, *F*(1, 4) = 6.51, *p* = .06, *r*^2^ = .62. However, there was a significant interaction effect between tempo condition and gender, *F*(10, 40) = 3.40, *p* < .01. As can be seen in Fig. 2, although for both males and females running cadence increased (or decreased) with increases (or decreases) in music tempo, these adjustments were more pronounced for women than for men. In addition, there was no significant effect of musical training, *F*(1, 4) = 6.48, *p* = .06, *r*^2^ = .62, which indicated that participants without musical training displayed similar levels of cadence adaptation as participants with a musical background. Finally, no significant interaction effect was found between musical training and tempo condition, *F*(10, 40) = 1.79, *p* = .10 (see Fig. 3).

### Entrainment Basin

**Fig. 4:**
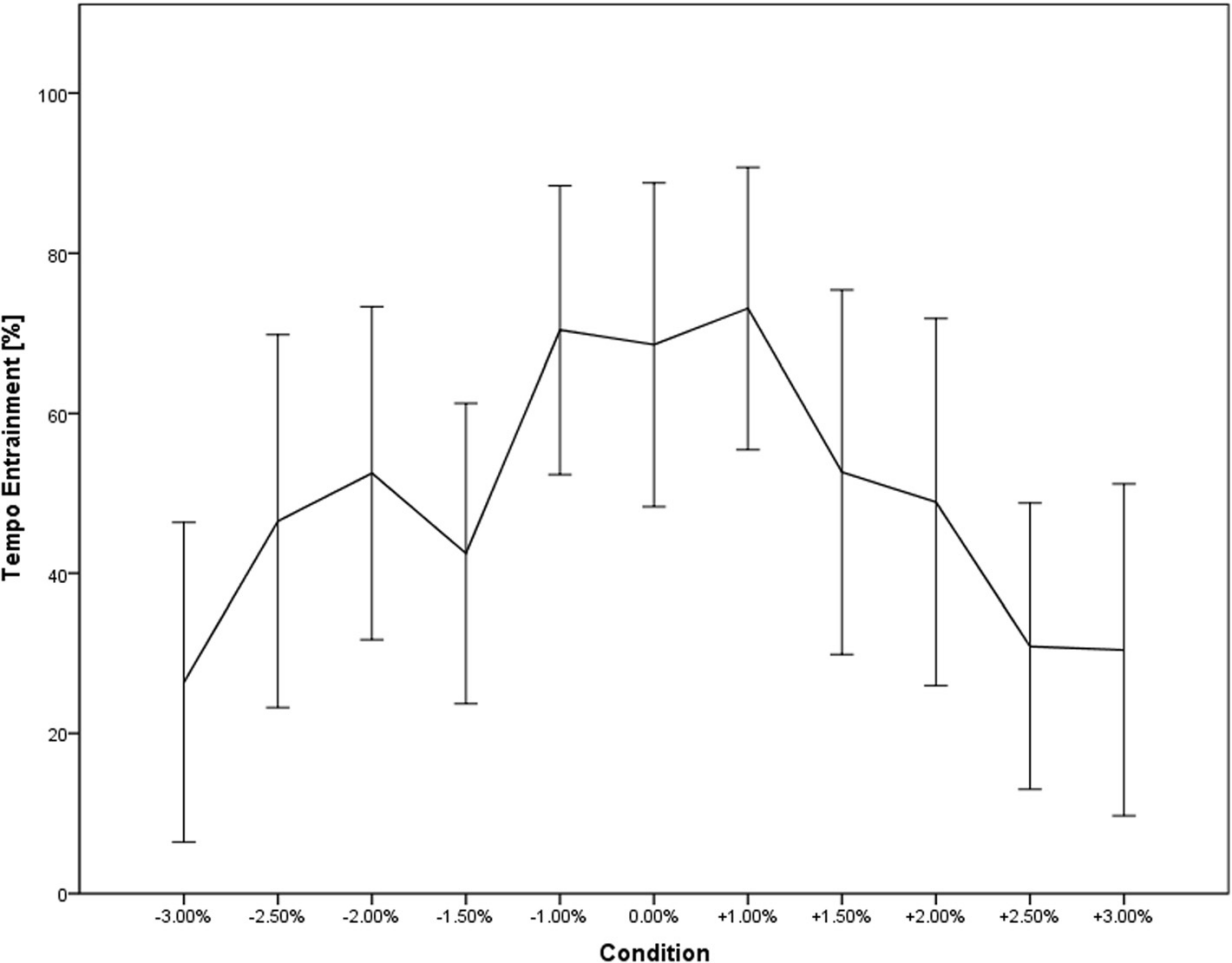
Entrainment basin displaying mean tempo entrainment for the different conditions. Data presented is mean ± SE

In order to trace a possible basin for entrainment, the effect of the conditions on the level of tempo entrainment was tested. KS tests showed that the entrainment values were significantly non-normal, *D*(161) = 0.15, *p* < .001. Friedman’s ANOVA showed a significant effect of condition on tempo entrainment, *χ*^2^(10) = 19.27, *p* < .05. Wilcoxon tests were used to follow up this finding, and all conditions were compared against the control condition (0 % of tempo change). A Bonferroni correction was applied, and all effects are thus reported at a .005 level of significance. It appeared that, compared to the control condition (Median (Mdn) = 74.25), tempo entrainment was significantly lower in the +2.50 % condition ((Mdn = 12.48), *Z* = −2.92, *r*^2^ = .53) and tended to be lower in the +3.00 % ((Mdn = 14.01), *Z* = −2.41, *p* = .016, *r*^2^ = .36) and −3.00 % conditions ((Mdn = 6.97), *Z* = −2.48, *p* = .013, *r*^2^ = .38). Figure 4 represents the mean tempo entrainment for every single condition.

**Fig. 5:**
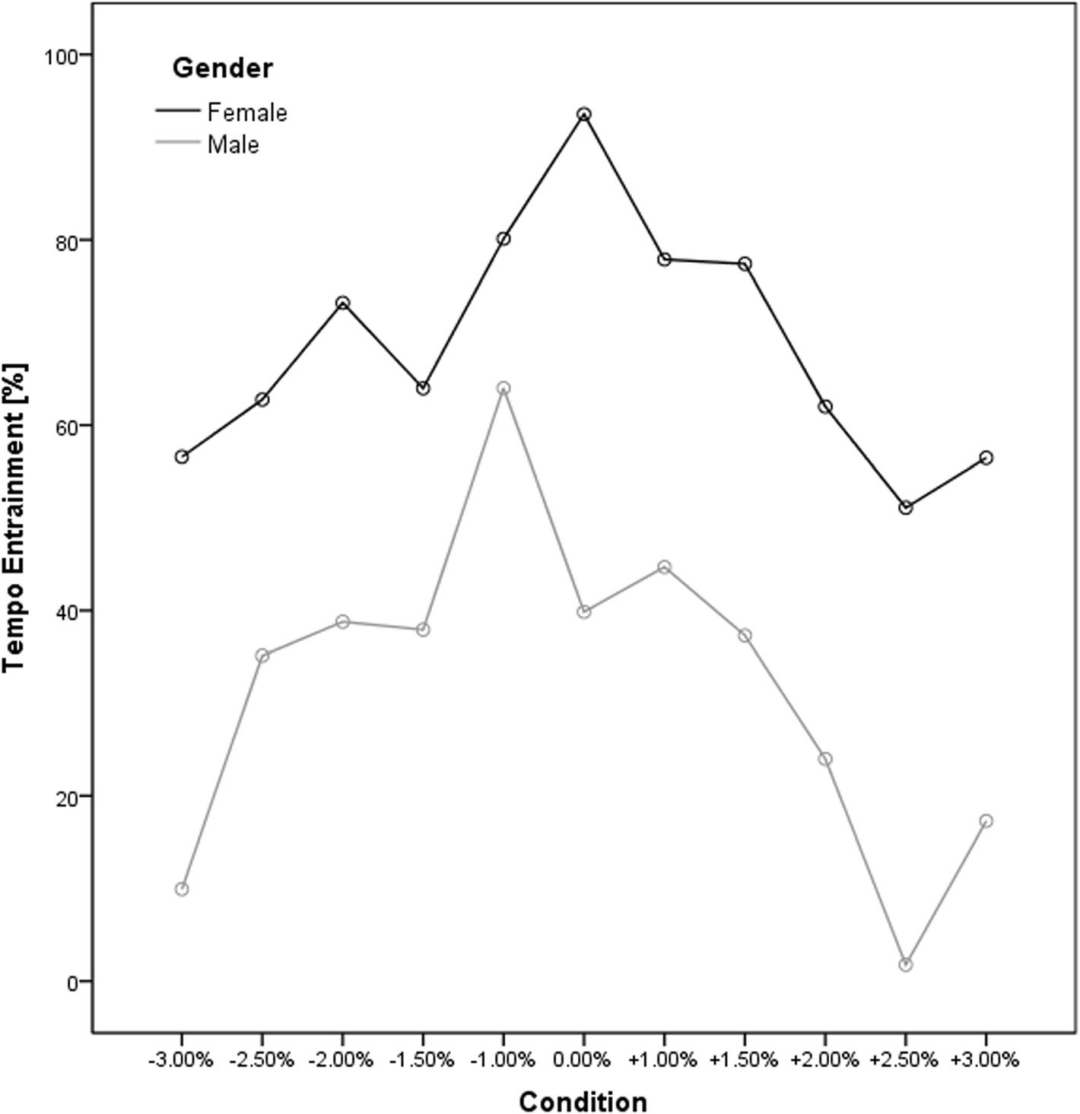
Interaction plot of estimated marginal means calculated for tempo entrainment at both gender levels

It is noteworthy that the entrainment basin did not differ significantly between females and males (see Fig. 5). However, the mean level of entrainment appeared to be higher for females as compared to their male counterparts. When testing this assumption, a Mann-Whitney test indeed revealed significantly higher levels of tempo entrainment for female participants (Mdn = 60.05) compared to their male counterparts (Mdn = 39.10), *U* = 10.00, *Z* = −2.28, *p* < .05, *r*^2^ = .32. It was also tested whether a link between musical training and entrainment could be found. However, no significant difference was found between participants with (Mdn = 50.73) or without musical background (Mdn = 38.24) regarding their level of entrainment, *U* = 18.00, *Z* = −1.30, *p* = .19, *r*^2^ = .11.

### Perceived Exertion

It was also checked whether the level of entrainment could be related to the degree of perceived exertion. For this purpose, a two-tailed Spearman’s correlation test was performed on entrainment values and ratings on the RPE scale. However, no significant relationship between perceived exertion and entrainment was found, *r*_s_ = −.04, *p* = .58.

## Discussion

The aim of this study was to examine whether music tempo could serve as a means to influence running cadence. Results indeed unveiled a significant relationship between imperceptible alterations in music tempo, in proportion to recreational runners’ self-paced running cadence, and cadence adaptation. In other words, faster music resulted in an increase, while slower music led to a decrease in running cadence. This effect can be explained through the idea of a sensorimotor mechanism that aligns footfall to musical beats. Adjustment of the footfalls to the beats relies on a phase-error correction mechanism of expected sensory outcomes [52]. Consequently, our study confirms results of previous research stressing the effect of music tempo on exercise performance [8, 11, 41, 53, 54]. This particular study also extends preceding research, as in this case, the effect on running cadence was tested using imperceptible changes in musical tempi with no explicit instructions regarding entrainment with the music. In contrast, in past research, participants were generally instructed to couple movement to music. Even if this was not the case, employed tempo variations usually proved to be too large to be unnoticeable. For example, Waterhouse, Hudson, and Edwards [41] compared cycling performance to normal, fast (increase of 10 %), and slow music (decrease of 10 %). Edworthy and Waring [8] examined treadmill-running behaviour when listening to music with a tempo of either 200 or 70 BPM, while Karageorghis et al. [53] employed tempi of 80, 120, and 140 BPM in their study on walking. In contrast, a maximum deviation of 3 % from the original music tempo was implemented in this particular study, as the amount of variation in tempo that an average person can distinguish is situated around 4 % [51]. Consequently, novel insights were presented in this study, as it was shown that recreational runners are able to adapt their running cadence (up to 2 % of the original cadence) to tempo changes in music (up to 3 % of the original tempo) without being aware of this attunement and without being instructed to do so. This finding supports the notion that an individual tends to synchronize spontaneously to an auditory rhythm occurring in the environment [37, 39, 52] and is in agreement with the natural predisposition of humans to respond to rhythmical qualities of music [34, 35].

It was also tested whether a basin for spontaneous entrainment of running cadence to music tempo could be found. Previous research has suggested that a range of period differences exists over which entrainment of movements of an individual with an environmental rhythm generally occurs and that beyond this range the occurrence of unintentional coordination is highly unlikely [33, 36–40]. Results indeed revealed a significant decrease in the level of entrainment in combination with increasing deviations from the original music tempo. The degree of entrainment with the tempo of the music dropped significantly as soon as tempo increases of 2.50 % were introduced but also tended to drop at decreases of 3.00 %. This could be explained by the fact that when deviations (especially increases) from the original, self-selected, and thus comfortable running tempo got larger, the effort required from the runner increased and at a certain point probably required too much effort, resulting in significantly lower levels of entrainment. As such, our results are in line with the idea of an entrainment basin for spontaneous coordination [33, 36–40]. However, our findings also contrast with those of Mendonça et al. [54], showing that for uninstructed synchronization of walking to music, participants did not adapt their step frequency to music that differed 5 to 10 % above and under their nominal step frequency, while they did adjust when synchronization was instructed. This could imply that a wider basin might be found for instructed entrainment to music tempo, while spontaneous entrainment occurs only when smaller deviations from the original tempo are introduced. But this is subject to some speculation and might benefit from further research.

Music is believed to only successfully distract from fatigue and discomfort when physiological awareness and perceived exertion are relatively low [19, 20, 22–24]. Therefore, in order to control for possible effects of perceived exertion, after each set of four laps, a break of approximately 5 min was introduced. Besides, the relationship between the degree of perceived exertion and the level of entrainment was also examined in the analysis. Nevertheless, no significant relationship between perceived exertion and entrainment was found. This could be due to the fact that, in general, participants did not perceive the task as extremely light or exceptionally hard but mostly rated their perceived exertion as intermediate. A reason for this might be that runners ran at their comfort tempo and no large shifts in the tempo of the music were incorporated in the study, but it might also be partly due to the introduction of the breaks after each condition. Besides, most previous research demonstrating decreasing levels of influence of music on attentional processes at higher exercise intensities tested this effect using asynchronous music, e.g. [19, 20, 22–24]. Whether this also applies to synchronous music still remains rather unclear, although, in their study on the effect of synchronous music on treadmill running, Terry et al. [29] did indicate lower levels of perceived exertion, assessed at moderate-to-high work intensities, for synchronous music compared to the no-music control. Yet, the magnitude of the differences in rating of perceived exertion proved to be rather small.

Another hypothesis referred to gender. We expected female participants to exhibit larger levels of entrainment in comparison with their male counterparts. Indeed, significantly higher levels of tempo entrainment were observed for females. In addition, although the effect of the music tempo on running cadence was unveiled for both males and females, changes in running cadence as a result of deviations in music tempi were more pronounced for female runners than for male ones, which suggests that women were more influenced by tempo changes than men. These findings resonate with the general belief that women are more responsive to musical stimuli [11, 41, 34, 55].

One should bear in mind that the current study focused on self-paced running, and thus, the type of exercise under study concerned one that is of low-to-moderate intensity. When studying activities with higher levels of intensity, music might not have a comparable effect on the exercisers’ performance, as when high workloads are undertaken, the exerciser’s attention could be shifted towards the painful or fatiguing effects of the exercise [19, 20, 22–24]. However, although most previous research on high-intensity exercise did not show any remarkable effects of music tempo, exemplary studies that have unveiled such effects do exist as well. In a study by Rendi, Szabo, and Szabo [10], for example, where exercisers were asked to perform a 500-m rowing sprint, in which physiological awareness is high, it was shown that fast-tempo music increased arousal and, in turn, performance, even during high-intensity sprints, while music with a slow tempo did not generate such stimulating effects. Further exploration of the impact of music tempo on sport activities with high workloads would be beneficial.

It could also be questioned whether spontaneous, thus uninstructed, entrainment is generally more beneficial with regard to exercise performance than instructed entrainment. It could be suggested that when synchronization is spontaneous, it may require less attentional resources, thus leading to even more important benefits (e.g. leaving free attentional resources to realize other tasks). Besides, exercise training could be simplified when instruction would prove to be redundant. On the other hand, it has been indicated that instructed synchronization is a form of active attentional manipulation, which has been shown to have more positive effects, at least in the form of perceived exertion and exercise efficiency [12, 28]. However, as this question has not been solved yet, the discussion whether spontaneous synchronization is more beneficial compared to instructed (or even imposed) synchronization should be unravelled in future studies.

In this particular study, recreational runners were tested. However, since music is believed to be more beneficial for recreational compared to trained exercisers [56], different results might have been obtained if competitive runners were tested. Previous research on treadmill running indicated that less trained exercisers might depend to a greater extent on the positive feeling states generated by music, while trained exercisers generally tend to focus on the tasks and specifics of their training [57, 58]. Furthermore, as (either recreational or professional) runners do not typically tend to run distances of 800 m consecutively, interrupted by short brakes, it might be interesting to investigate whether the effect of music tempo is sustained over the course of longer, interrupted distances. Whether the entrainment basin for recreational runners would differ from that of professional runners and whether its effects are sustained over longer distances could be tested in future research.

## Conclusions

To conclude, it was unveiled that music tempo could serve as an unprompted means to re(shape) running cadence of recreational runners. This influence was shown to have a certain range, which suggests that maximal effects of music tempo can only be obtained up to a certain level of tempo change and proved to be stronger for female compared to male runners. As modifying step rate may prove beneficial in the prevention and treatment of common running-related injuries, this novel finding could be especially relevant for treatment purposes, such as exercise prescription and gait retraining.

## Additional file

Additional file 1:
**Validity and reliability of the testing equipment.** During development of the D-Jogger system, several tests/experiments/pilots were conducted to verify and correct individual components and the final device (D-Jogger system + iPod sensors).
