# Erratum to: Spontaneous Entrainment of Running Cadence to Music Tempo

**DOI:** 10.1186/s40798-015-0030-z

**Published:** 2015-09-11

**Authors:** Edith Van Dyck, Bart Moens, Jeska Buhmann, Michiel Demey, Esther Coorevits, Simone Dalla Bella, Marc Leman

**Affiliations:** 1 IPEM, Department of Arts, Music and Theatre Sciences, Ghent University, Technicum Blok 2, Sint-Pietersnieuwstraat 41, Ghent, Belgium; 2 EuroMov, Movement 2 Health Laboratory (M2H), University of Montpellier, 700 Avenue du Pic Saint Loup, Montpellier, France

Unfortunately, the original version of this article [1] contained an error. In the captions for both Fig. 1 and Fig. 4, the sentence "Data presented is mean ± SE" should be replaced by "Data presented is mean ± 95 % CI" as presented below:

**Fig. 1:**
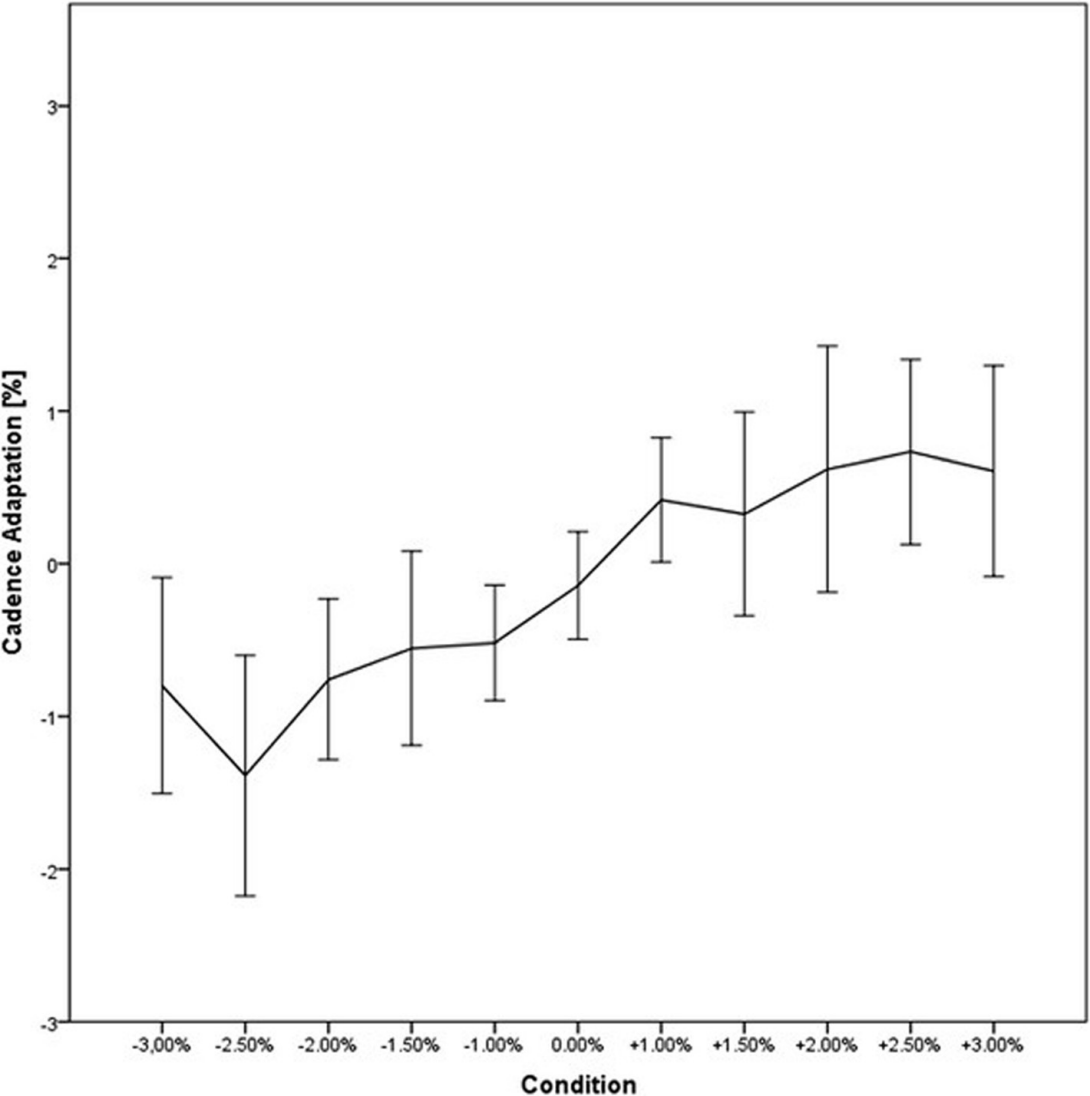
Mean tempo and cadence adaptation for the different conditions. Data presented is mean ± 95 % CI

**Fig. 4:**
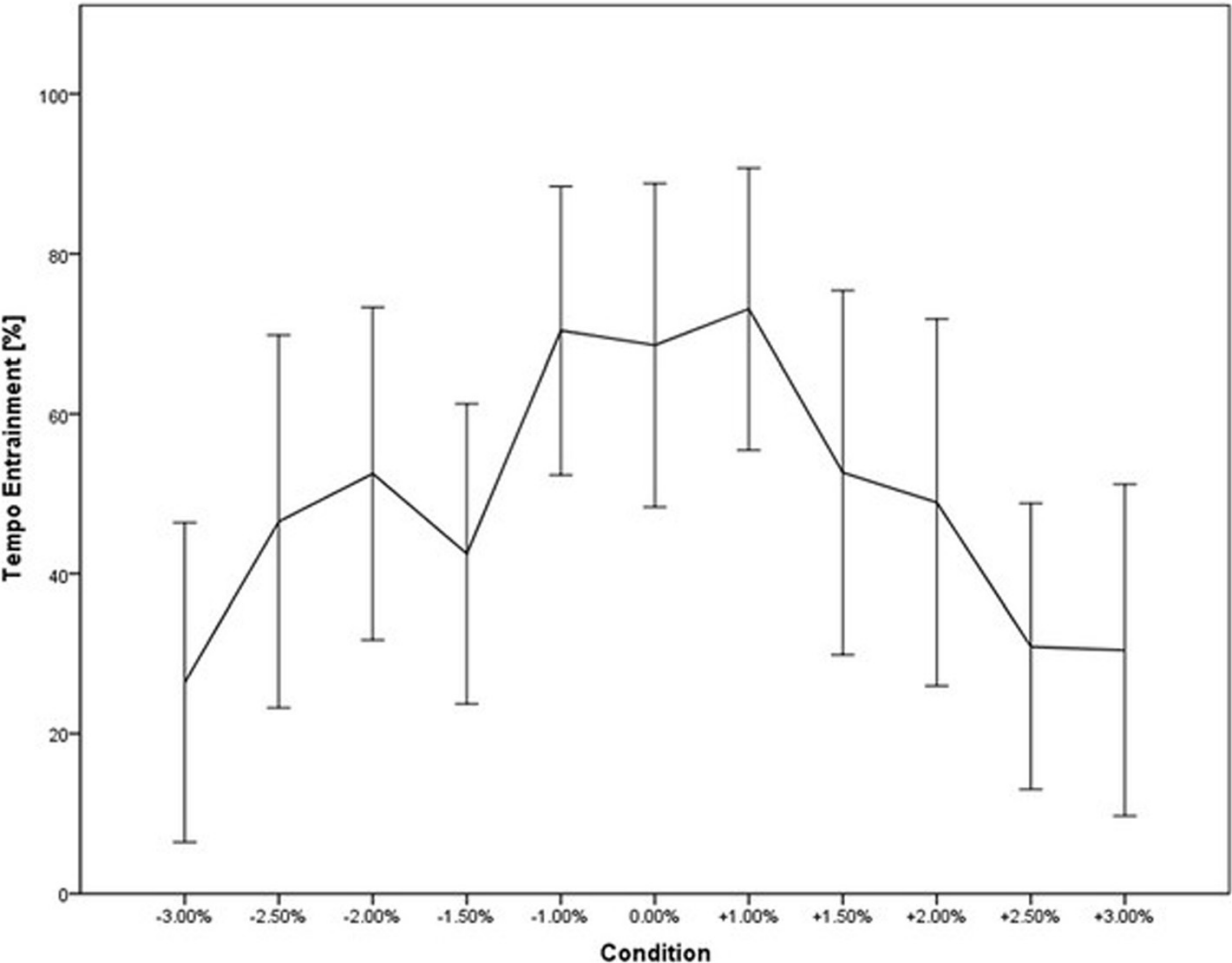
Entrainment basin displaying mean tempo entrainment for the different conditions. Data presented is mean ± 95 % CI
