# Supplementary material for: Spontaneous Entrainment of Running Cadence to Music Tempo
Source: Sports Med Open. 2015 Jul 14;1:15. doi: 10.1186/s40798-015-0025-9 (PMC4526248; doi:10.1186/s40798-015-0025-9)
Supplement: Additional file 1: — Validity and reliability of the testing equipment. During development of the D-Jogger system, several tests/experiments/pilots were conducted to verify and correct individual components and the final device (D-Jogger system + iPod sensors). [file 40798_2015_25_MOESM1_ESM.docx]

**Validity and reliability of the testing equipment**

During development of the DJogger system, several tests/experiments/pilots were conducted to verify and correct individual components and the final device (DJogger system + iPod sensors).

**1) Validity of the sensor data**

Initially, more professional data capture systems were used, such as the Xsens (9DOF sensors, wired to central hub on body, wireless transfer to pc), mobility lab (see below) and the Delsys wireless accelerometer and gyroscope system. However, they all are expensive, cumbersome to setup (especially in ecological settings - one of our main aims) and required larger hardware to be attached to the participant.

After looking for alternatives, the decision was made to opt for iPods as they have some great usability features: long battery life, integrated sensors + streaming apps, not too expensive, easy to replace (it would not be the first one to fall/break/get moist due to sweat) and comfortably attachable using specifically designed third party pockets for runners (albeit for use on their upper arm).

Prior to using the iPods, they were compared in a synchronous data capture experiment to the Delsys accelerometer and gyroscope system. The key results were:

- Signals from all sensors agree well
- Delay between sensors' arrival time on the receiving windows platform is minor (10 ms maximum) in optimal conditions:
  - The Wi-Fi hotspot uses a free Wi-Fi channel (no other networks in the same range)
  - No 2.40 gHz interference nearby
  - Distance smaller than 10 m
- Up to 60 m no samples were lost but jitter increased (inter-sample time variation increased; resulting in the use of a receiving buffer).
  - A direct line of sight between sender (iPod) and receiver (hotspot) at distances greater than 10 m is required to minimize jitter

The following plot shows a comparison between Delsys systems (IM2&4) and the iPod systems for both gyroscope and accelerometer.


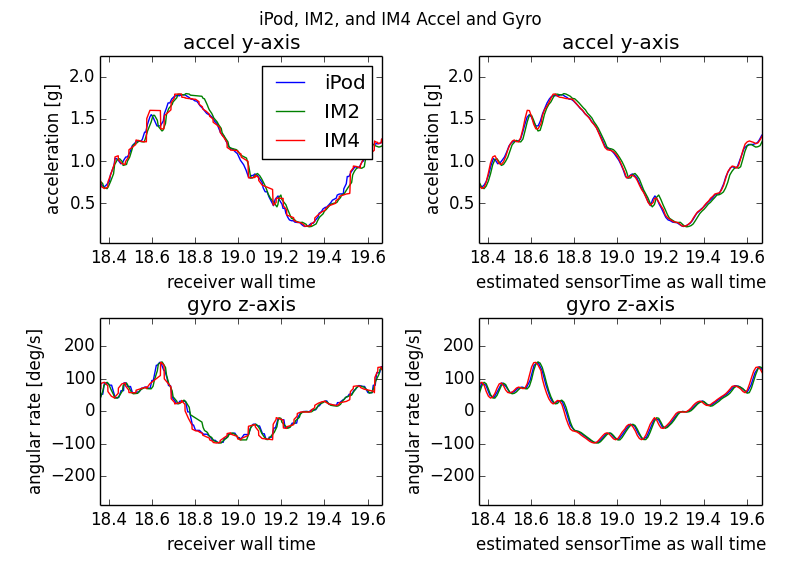


**2) Validity of the step detection**

For verifying the step detection algorithm, we coupled the DJogger system with Mobility Lab; a specialized software and hardware suite for wireless gait analysis focused on rehabilitation. The system is verified and frequently used in scientific experiments.

The following phase histogram shows the difference in timings of a detected step in DJogger and a detected step in Mobility Lab. It is clear that both systems detect steps at approximately the same time, with a mean phase angle difference of 6° (~ 8 ms at 120 SPM). Given the consistency of these results, the step detection algorithm was deemed acceptable for real-time feedback.


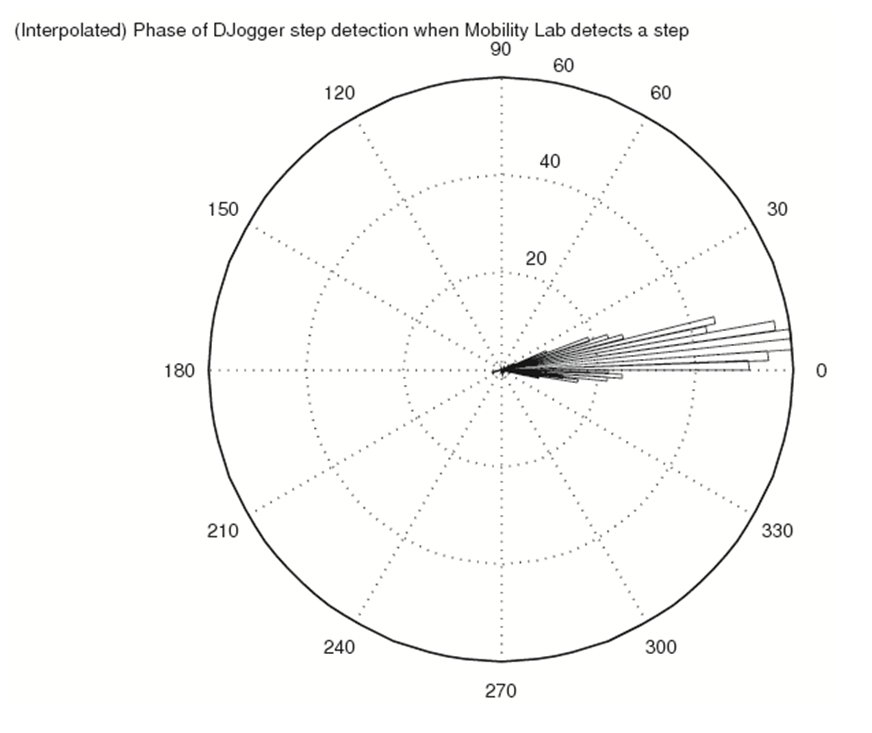


More info about mobility lab:

- http://apdm.com/gait-and-posture/Mobility-Lab/
- http://www.ncbi.nlm.nih.gov/pmc/articles/PMC4062543/

**3) Audio latency tests**

Sound engines/drivers and hardware on computers all have a certain delay or latency due to buffering and operating system implementation. When the request is made to 'play a sound', the user does not perceive it immediately.

The system was optimized to minimize this lag using ASIO drivers, with fixed buffers of 128 samples and a playback rate of 44.10 kHz (stereo). This means the latency of the sound hardware is theoretically 5.80 ms. The software system itself, written in Max MSP (focused on real-time audio) also adds some delay due to DSP components (e.g., the phase vocoder). Max MSP features a tool for measuring the total latency of such systems (by connecting the line out to the line in of the computer and sending test signals while measuring the time).

Our total latency fluctuated for the output line between 8 and 10 ms.

**4) DJogger verification: Response time**

Finally, when putting all components together (DJogger), an additional verification had to be performed to verify the step detection auditory feedback. The aim was to answer one main question: Is the auditory feedback of a detected step perceived at the actual footfall? The DJogger system was configured to play a beat as soon as a step was detected (i.e. at 0° phase).

Measurements were taken using a separate Delsys system:

- FSR (pressure) sensors were embedded in the participant's shoe sole at the heel for measuring ground reaction force (GRF). These were seen as the 'ground truth' for step detection: The moment of a heel strike is very easily detected.
- IPods were used to stream the accelerometer and gyroscope data to the computer for the step detection. Audio from the computer was connected to the 2000 Hz ADC of the Delsys system. A simple impulse beat was played back each detected step.

This way, a simultaneous data capture of a running participant was made for both ground truth FSR sensors and the perceived audio (i.e. auditory feedback on a footfall).

The following graph shows a sample of the received data:


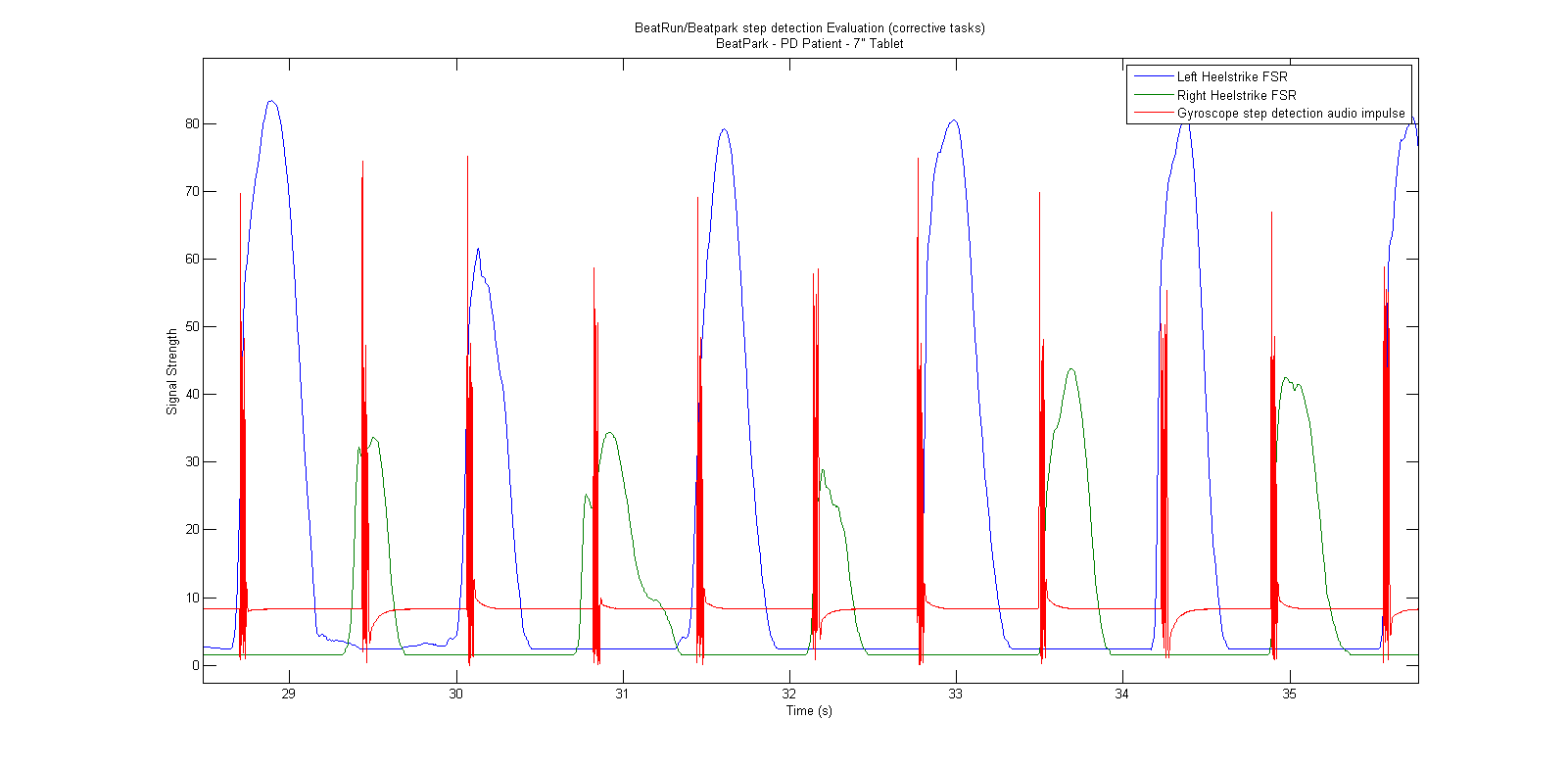


General results show the start of the audio feedback (red line) starts at 16 ms (+- 8 SD) after the onset of the heel strike. This coincides with the 8 ms delay for the step detection (compared to mobility lab) + 8-10 ms delay of the audio system.

Because the total delay or response time was consistent around 16 ms, we accepted the validity of the Djogger prototype with iPods in terms of step detection, phase calculations and real-time auditory feedback.

As a comparison, the natural sound of the onset of a heel strike reaches one's ear around 5.26 ms (1.80 m at 343 m/s)

**5) DJogger algorithm verification**

Please check:

Moens B, Muller C, van Noorden L, Franěk M, Celie B, Boone J, Bourgois J, Leman M. Encouraging spontaneous synchronization with D-Jogger, an adaptive music player that aligns movement and music. Plos One. 2014;doi: 10.1371/journal.pone.0114234
